# Novel snake papillomavirus does not cluster with other non-mammalian papillomaviruses

**DOI:** 10.1186/1743-422X-8-436

**Published:** 2011-09-12

**Authors:** Christian E Lange, Claude Favrot, Mathias Ackermann, Jessica Gull, Elisabeth Vetsch, Kurt Tobler

**Affiliations:** 1 Dermatology Department, Clinic for Small Animal internal Medicine, Vetsuisse Faculty, Winterthurerstrasse 260, CH-8057 Zurich, Switzerland; 2 Institute of Virology, Vetsuisse Faculty, Winterthurerstrasse 266a, CH-8057 Zurich, Switzerland; 3 Clinic for Zoo Animals, Exotic Pets and Wildlife, Vetsuisse Faculty, Winterthurerstrasse 260, CH-8057 Zurich, Switzerland

## Abstract

Papillomaviruses (PVs) are associated with the development of neoplasias and have been found in several different species, most of them in humans and other mammals. We identified, cloned and sequenced PV DNA from pigmented papilloma-like lesions of a diamond python (*Morelia spilota spilota*). This represents the first complete PV genome discovered in a Squamata host (MsPV1). It consists of 7048 nt and contains the characteristic open reading (ORF) frames E6, E7, E1, E2, L1 and L2. The L1 ORF sequence showed the highest percentage of sequence identities to human PV5 (57.9%) and Caribbean manatee (*Trichechus manatus*) PV1 (55.4%), thus, establishing a new clade. According to phylogenetic analysis, the MsPV1 genome clusters with PVs of mammalian rather than sauropsid hosts.

## 

The members of the family *Papillomaviridae *are non-enveloped, icosahedral viral particles with a diameter of 50 to 55 nm and a small (~8 kbp), circular, double stranded DNA genome, which is transcribed into one single direction [1]. The family may be divided into at least 29 genera with a vast number of species, types, subtypes, and variants of papillomaviruses (PVs), based on nucleotide identities of the L1 open reading frames (ORFs) [2]. Due to the high genetic diversity and the host range it is anticipated, that the family *Papillomaviridae *has a long evolutionary history, but details remain yet vague.

DNA of PVs can be detected in a wide range of vertebrate species, thus far including humans, various other mammals, birds, and turtles (additional file 1). PVs have been found to play a role in several human diseases of the skin and mucous membranes. The DNA of PVs can be amplified from samples of clinical asymptomatic individuals, but more importantly their influence on the development of certain benign and malignant disorders was demonstrated repeatedly [3,4].

The sequences of three PVs from birds, namely the Chaffinch (*Fringgilla coelebs*), the Yellownecked Francolin (*Francolinus leucoscepus*) and the Timneh African gray parrot (*Psittacus erithacus timneh*), have been determined [5-7]. Various data suggest the existence of sauropsid-specific PVs in association with papillomas in lizards, snakes, crocodiles and turtles [8-11]. Sequences of the entire genome from two PVs of turtles were determined recently from the Loggerhead turtle (*Caretta caretta*) and the Green seaturtle (*Chelonia mydas*) [12]. Upon phylogenetic analysis, these five sauropsid PVs cluster together and appear clearly distinct from the PVs infecting mammalian species.

Here, we report on the cloning, sequence determination and phylogenetic analysis of a PV-specific DNA from the Australian diamond python (*Morelia spilota spilota*).

Samples from a diamond python with small black papillated and pedunculated exophytic skin proliferations were taken and stored at -20°C until processing.

Total DNA from a 25 mg tissue sample was isolated using a QIAamp DNA extraction kit (Qiagen) according to the manufacturer's recommendations. One microliter of the extracted DNA was used for RCA [13], using a TempliPhi Amplification kit (General Electrics Biosciences). Slight modifications were applied to the protocol supplied by the manufacturer: 1 μl of 10 mM dNTPs was added and the reaction time was prolonged to 16 h at 30°C. Amplified DNA was cloned into the EcoRI or XhoI site of pBluescript II KS+ (Stratagene) using standard procedures.

The nucleotide sequence of cloned DNA and of precipitated RCA product was determined (Microsynth) on both strands by cycle sequencing using an ABI 377 sequencer (Applied Biosystems). The primary sequences were assembled using Contigexpress software (Vector NTI Informax).

The novel sequence was compared with published PV sequences including: Moose (*Alces alces*): AaPV1 (M15953), bovine (*Bos primigenius*): BPV1 (X02346), BPV3 (NC_004197), BPV5 (AF457465), BPV7 (NC_007612), marine turtles: *Caretta caretta*: CcPV1 (EU493092) and *Chelonia mydas*: CmPV1 (EU493091), caprine (*Capra hircus*): ChPV1 (NC_008032), canine (*Canis lupus familiaris*): CPV1 (L22695), CPV2 (AY722648), CPV3 (NC_008297), CPV4 (NC_010226), equine (*Equus caballus*): EcPV1 (AF498323), EcPV2 (NC_012123), porcupine *(Erethizon dorsatum*): EdPV1 (NC_006951), hedgehog (*Erinaceus europaeus*): EePV1 (EF396272), birds (*Fringilla coelebs*): FcPV1 (NC_004068), *Francolinus leucoscepus*: FlPV1 (EU188799) and *Psittacus erithacus*: PePV1 (NC_003973), feline (*Felis catus*): FdPV1 (NC_004765), FdPV2 (EU796884), human (*Homo sapiens*): HPV1 (NC_001356), HPV4 (NC_001457), HPV5 (NC_001531), HPV6 (NC_001355), HPV9 (NC_001596), HPV16 (FJ610146), HPV18 (NC_001357), HPV41 (NC_001354), HPV49 (NC_001591), HPV50 (NC_001691), HPV63 (NC_001458), HPV88 (NC_010329), HPV92 (NC_004500), hamster: *Mesocricetus auratus*: MaPV1 (E15111), non-human primate (*Macacca mulata*): MmPV1 (NC_001678), muridae (*Micromys minutus*): MmiPV1 (DQ269468) and (*Mastomys natalensis*): MnPV1 (NC_001605), ovine (*Ovis aries*): OaPV1 (NC_001789), rabbit (*Oryctolagus cuniculus*): OcPV1 (NC_002232), racoon (*Procyon lotor*): PlPV1 (NC_007150), Porpoise (*Phocoena spinipinnis*): PsPV1 (NC_003348), fruit bat (*Rousettus aeyptiacus*): RaPV1 (NC_008298), cottontail rabbit (*Sylvilagus floridanus*): SfPV1 (NC_001541), porcine (*Sus scrofa*): SsPV1 (NC_011280), manatee (*Trichechus manatus*): TmPV1 (NC_006563), dolphin (*Tursiops truncatus*): TtPV1 (EU240894), TtPV2 (NC008184) and polar bear (*Ursus maritimus*): UmPV1 (NC_010739). The coding sequences for the E1, E2, L2 and L1 proteins from those fifty papillomaviruses including MsPV1 and the 5 previously published sauropsoid PVs were aligned on the amino acid level by using the Perl script transAlign.pl [14]. This script translates the nucleotide sequences to amino acid sequences and passes them to ClustalW [15]; (version 2.0.10, Gonnet matrix, default settings) before back-translate to DNA sequences. The four sets of aligned nucleotide sequences representing the four sets of protein sequences were combined to a single multiple sequence alignment (MSA) by concatenating the sequences from each virus. The resulting single MSA, 8709 nucleotide positions in length, was then shortened to 5100 nucleotide positions by using GBlock (version 0.91b; half gap positions allowed [16]). The optimal model of DNA evolution was evaluated for best fit of the data set using ModelTest (version 1.4.4; default settings [17]). Bayesian phylogeny was inferred using MrBayes (version 3.2; Markov Chain Monte Carlo (MCMC) with GTR substitution matrix, variable gamma rates, invariant sites, two runs four chains of 1'000'000 generations), and displayed with FigTree (1.3.0) [18]. The MCMC was sampled every 1'000 cycles. Stationarity was reached after less than 10'000 cycles according to Tracer analysis [18]. The tree was midpoint rooted. Pairwise alignments were made in Needle (Emboss) using the ednafull matrix with a gap penalty of 10.0 and a extend penalty of 0.1. The nucleotide sequence data of the PV was deposited in GenBank under accession no. HQ262535.

Analyses of the cloned sequence confirmed that papillomavirus DNA had actually been detected. The amplified genome consists of 7048 bp and has a GC content of 41%. ORFs putatively encoding E6, E7, E1, E2, E4, L2 and L1 but no E5 were identified (Figure 1). Deduced amino acid sequences of the putative proteins revealed a degenerate ATP-dependent helicase motif GQPNTGKS in E1, two putative metal-binding motifs (CX_2_CX_29_CX_2_C) in E6, one such motif in E7, and one pRb binding domain (LXCXE) [19,20]). The deduced amino acid sequences of both structural proteins L1 and L2 are predicted to harbour a basic tail at their C termini.

**Figure 1:**
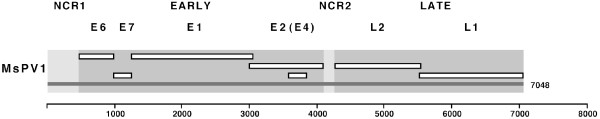
**Schematic presentation of the *Morelia spilota spilota *papillomavirus genome and open reading frames (ORFs)**. Genomes are divided into sections: early genes (Early), late genes (Late) and non-coding regions (NCRs). Numbers indicate nucleotide positions. Nucleotide position number one is defined here as the first following the stop codon of the L1 ORFs.

A non coding region (NCR1) between the stop-codon of the L1 ORF and the start-codon of the E6 ORF was 473 nt in length. A second non coding region (NCR2) of 178 nt was between the stop-codon of the E2 and the start-codon of the L2 ORF.

In addition, papillomavirus-specific DNA motifs were identified in the readily determined genome sequence. Four putative consensus sequences for E2 binding (ACCN_5-7_GGT) were detected; two of these were located in the NCR1 (positions 6919-6931 and 32-43) and two were located within the predicted L1 ORF (positions 5936-5948 and 5990-6000). Within the NCR1, a putative origin of DNA replication was identified, consisting of two E2-binding regions flanking a region with 54% A/T content. Two polyadenylation consensus sequences (AATAAA) were predicted, one within the NCR1 (position 6729-6734) and the other within the NCR2 (position 3816-3821).

In order to possibly allocate the novel PV in the evolutionary context, phylogeny based on the aligned E1-E2-L2-L1 sequences was determined. Sequences of fifty PVs, representing all presently classified genera and MsPV1 were included in these analyses (Figure 2). While all other sauropsid PVs clustered together, MsPV1 was located far from any of them. Interestingly, MsPV1 was found in relative proximity to the PV (TmPV1) of a marine mammal, the manatee (sea cow).

**Figure 2:**
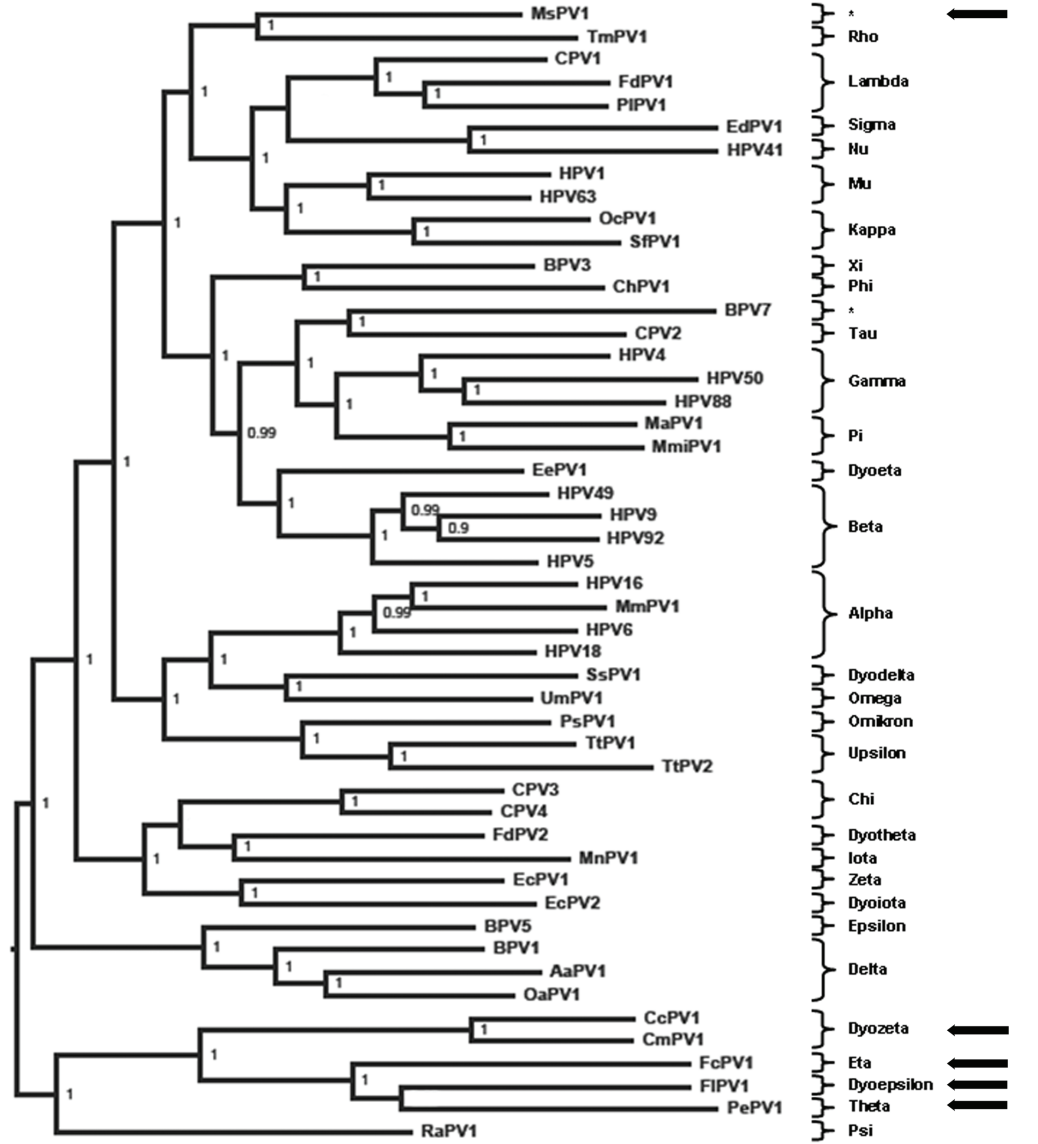
**Midpoint rooted Bayesian phylogenetic tree of 50 papillomaviruses (PVs)**. Numbers at internal nodes represent the posterior probability support values; values below 0.9 are not shown. Unclassified PVs are marked with asterisks. Arrows indicate sauropsid PVs.

Furthermore, Needle alignments which reflect global alignments of the entire genes in contrast to the alignment used for the tree were made. Upon these pairwise alignments on the nucleotide level of the L1 ORF the highest percentage of sequence identities was found with a human PV (HPV5; 57.9%) and with TmPV1 on the E1 ORF (55.6%) (Table 1). Consequently MsPV1 may be regarded as rather a close to the root prototype of a new PV clade that might establish a new genus regarding to the guidelines of PV classification [2,21].

**Table 1:** Comparison of MsPV1 to selected PVs

ORF	CcPV1	CmPV1	FcPV1	FlPV1	PePV1	TmPV1	HPV5
E6	33.3	33.4	-	33	-	39.8	38.8

E7	39.3	36.1	25.3	31.3	-	37	38.1

E1	45.1	47.3	41.6	44.4	45	55.6	52.9

E2	38.7	40.3	37.8	36.6	44.9	43.5	40.7

L2	38.7	37.6	37.4	35.3	36	36.8	39.3

L1	45.5	46.4	49	47.3	46.3	55.4	57.9

Pairwise comparison of the open reading frames of the MsPV1 to a range of selected PVs on the nucleotide level indicated as percentage of sequence identities.

While many PVs have been described in humans and other mammalian species, the number of known PVs infecting non-mammalian species is limited (additional file 1). The described MsPV1 genome represents the first PV isolated from a snake. It contains the characteristic ORFs E6, E7, E1, E2, L1 and L2, a large non-coding region between L1 and E6 as well as a small non-coding region between E2 and L2. The size of the viral genome (7048 bp) is comparable with the two turtle PV genomes (CcPV1; 7020 bp and CmPV1; 6953 bp), which are all relatively small. However, while the latter two viruses have short versions of E1, E2, L2 and E6 ORFs as well as the NCR, the small size of MsPV1 is primarily due to comparably short E2 and L2 ORFs and also comparably short NCRs.

The genomic sequences of the PVs from three bird and two turtle species have previously been published [5-7,12]. According to our and other's phylogenetic analysis, they cluster together. However, our newly discovered PV genome isolated from the snake did not at all cluster with the five other sauropsid PVs (CcPV1, CmPV1, FcPV1, FlPV1 and PePV1). Upon pairwise alignment of individual ORFs, MsPV1 shares much higher percentage of sequence identities with mammalian PVs than with any of the known sauropsid PVs (Table 1). This finding raises interesting questions in the context of PV evolution. While co-evolution with the host has been suggested and demonstrated to play a role in PV evolution [5,22,23] it has also been shown that PV evolution is probably a complex matter and other mechanism such as crossing of species barriers and adaptive radiation have to be considered as well [24-26].

Turtles as putative representatives of the Anapsida and snakes as representatives of the Diapsida are phylogenetically distinct with a common ancestor dating back more than 200 million years [27]. However, the three PVs isolated from birds (FcPV1, FlPV1 and PePV1), which also belong to the Diapsida, are phylogenetically much closer to the turtle PVs (CcPV1, CmPV1) than to MsPV1. As the snake PV appears closer to mammalian PVs, one explanation could be that it belongs to a lineage of PVs, which existed already in Amniota species, that lived before the split of Synapsida (mammals and mammal-like reptiles) and Sauropsida. The five other sauropsid PVs could under these circumstances go back to a second distinct ancestral lineage. However, an alternative explanation could be that some ancestor of this virus had been able to cross species barriers between the Sauropsida and the Synapsida, even long after their separation.

The limited number of available sauropsid PV sequences does not allow to precisely elucidate the ancient events. However, the sequence of this first PV genome found in a snake may be a valuable piece in the puzzle of the history of PVs.

## Competing interests

The authors declare that they have no competing interests.

## Authors' contributions

CEL did most of the molecular biological work, participated in the bioinformatic analysis and drafted the manuscript. CF participated in the coordination of the project and was involved in revising the manuscript. MA contributed to writing and revising the manuscript. JG collected the samples, and participated in revising the manuscript. EV was involved in molecular biological work. KT did most of the bioinformatic analysis and helped to draft the manuscript. All authors were involved in the planning of the experiments and have read and given final approve to the manuscript.

## Supplementary Material

Additional file 1 **Table Amniota**. Table of the main branches of extend Amniote orders. Simplified based on Benton MJ: The evolution of early amniotes. In *Vertebrate Paleontology*. 3rd edition. Oxford: Blackwell Publishing Ltd; 2005: 119-148. Click here for file
