# Supplementary material for: Novel snake papillomavirus does not cluster with other non-mammalian papillomaviruses
Source: Virol J. 2011 Sep 12;8:436. doi: 10.1186/1743-422X-8-436 (PMC3179961; doi:10.1186/1743-422X-8-436)
Supplement: Additional file 1 — Table Amniota. Table of the main branches of extend Amniote orders. Simplified based on Benton MJ: The evolution of early amniotes. In Vertebrate Paleontology. 3rd edition. Oxford: Blackwell Publishing Ltd; 2005: 119-148. [file 1743-422X-8-436-S1.PDF]

Additional file 1: Table Amniota

Amniota

	**Synapsida**			
		Mammalia		
			Monotremata	
			Didephimorpha	
			Paucituberculata	
			Microbiotheria	
			Notoryctemorphia	
			Dasyuromorphia	
			Peramelemorphia	
			Diprotodontia	
			Afrosoricida	
			Macroscelidea	
			Tubulidentata	
			Hyracoidea	
			Proboscidea	
			Sirena	(1)
			Cingulata	
			Pilosa	
			Scandentia	
			Dermoptera	
			Primates	(136)
			Rodentia	(4)
			Lagomorpha	(2)
			Eulipotypha	(2)
			Chiroptera	(1)
			Pholidota	
			Carnivora	(15)
			Perissodactyla	(2)
			Artiodactyla	(17)
			Cetacea	(4)
Sauropsida				
	**Anapsida**			
			Testudines	(2)
	**Diapsida**			
		Archosauria		
			Pinosauria	(3)
			Crocodylomorpha	
		Lepidosauria		
			Rhynchocephalla	
			Squamata	

Table of the main branches of extend Amniote orders. Simplified based on Benton MJ: The evolution of early amniotes. In *Vertebrate Paleontology*. 3rd edition. Oxford: Blackwell Publishing Ltd; 2005: 119-148.
